# Brain microRNAs associated with late-life depressive symptoms are also associated with cognitive trajectory and dementia

**DOI:** 10.1038/s41525-019-0113-8

**Published:** 2020-02-06

**Authors:** Thomas S. Wingo, Jingjing Yang, Wen Fan, Se Min Canon, Ekaterina Sergeevna Gerasimov, Adriana Lori, Benjamin Logsdon, Bing Yao, Nicholas T. Seyfried, James J. Lah, Allan I. Levey, Patricia A. Boyle, Julia A. Schneider, Philip L. De Jager, David A. Bennett, Aliza P. Wingo

**Affiliations:** 1 Department of Neurology, Emory University School of Medicine, Atlanta, GA USA; 2 Department of Human Genetics, Emory University School of Medicine, Atlanta, GA USA; 3 Department of Psychiatry, Emory University School of Medicine, Atlanta, GA USA; 4 Sage Bionetworks, Seattle, WA 98109 USA; 5 Department of Biochemistry, Emory University School of Medicine, Atlanta, GA USA; 6 Rush Alzheimer’s Disease Center, Rush University Medical Center, Chicago, IL USA; 7 Center for Translational and Computational Neuroimmunology, Department of Neurology, Columbia University Medical Center, New York, NY USA; 8 Division of Mental Health, Atlanta VA Medical Center, Decatur, GA USA

**Keywords:** Molecular medicine, Depression

## Abstract

Late-life depression is associated with an increased risk for dementia but we have limited knowledge of the molecular mechanisms underlying this association. Here we investigated whether brain microRNAs, important posttranscriptional regulators of gene expression, contribute to this association. Late-life depressive symptoms were assessed annually in 300 participants of the Religious Orders Study and Rush Memory and Aging Project for a mean of 7 years. Participants underwent annual cognitive testing, clinical assessment of cognitive status, and uniform neuropathologic examination after death. microRNAs were profiled from the prefrontal cortex using NanoString platform in the discovery cohort and small RNA sequencing in the replication cohort. A global microRNA association study of late-life depressive symptoms was performed using linear mixed model adjusting for the potential confounding factors. Four brain microRNAs were associated with late-life depressive symptoms at adjusted *p* < 0.05: miR-484, miR-26b-5p, miR-30d-5p, and miR-197-3p. Lower expression levels of these miRNAs were associated having greater depressive symptoms. Furthermore, lower levels of miR-484 and miR-197-3p were associated with faster decline of cognition over time. Moreover, lower miR-484 level was associated with higher probability of having Alzheimer’s dementia. Importantly, the associations between miR-484 and depressive symptoms and Alzheimer’s dementia, respectively, were replicated in an independent cohort. Lastly, the predicted targets of miR-484 were enriched in a brain protein co-expression module involving synaptic transmission and regulation of synaptic plasticity. This study identified four brain microRNAs associated with late-life depressive symptoms assessed longitudinally. In addition, we found a molecular connection between late-life depression and dementia through miR-484.

## Introduction

Late-life depression is associated with an increased risk for dementia, including Alzheimer’s disease (AD) and vascular dementia, with an odds ratio of 1.85 based on meta-analyses of 23 prospective studies including >49,600 participants.^1,2^ Notably, depression has been shown to be associated with faster decline of cognitive performance and with the clinical diagnosis of AD or vascular dementia but not with the traditional dementia pathologies, such as beta-amyloid plaques, tau tangles, Lewy bodies, hippocampal sclerosis, microinfarcts, and macroinfarcts.^3–6^ How depression and its associated molecular changes act to increase dementia risk is not well understood and is the focus of this work.

MicroRNAs (miRNAs) are important posttranscriptional regulators of gene expression and have been implicated in many neuropsychiatric disorders.^7–10^ miRNAs are sometimes referred to as “master regulators” because one miRNA can regulate hundreds of genes and thus can exert a substantial effect on the gene expression networks.^10^ A study of plasma miRNAs identified miR-184 to be associated with late-life depression.^11^ A systematic review gleaned plasma miRNAs associated with major depression and AD, respectively, from published studies and identified seven miRNAs common to both disorders.^12^ Other studies of brain miRNAs in major depression, though providing valuable information, either focused on candidate miRNAs or had relatively small sample sizes.^13^ Hence, studies of global brain miRNA profiles in depression with a larger sample size and with a focus on late-life depression are needed to provide more generalizable findings and insights into mechanisms by which depression increases risk for AD and vascular dementia.

To understand the molecular underpinnings behind detrimental effects of depression on dementia risk, we first performed a global miRNA association study of longitudinally assessed depressive symptoms in non-demented older adults who were followed for a mean of 7 years. We found four miRNAs significantly associated with late-life depressive symptoms. Next, we examined these four miRNAs in relation to the dementia-related traits, including rate of decline of cognitive performance over time, clinical diagnosis of mild cognitive impairment (MCI) or dementia, and eight measured neuropathologies. Two miRNAs were found to be associated with these dementia features (miR-484 and miR-197-3p). Then we examined the targets of these two miRNAs in a published protein co-expression networks and found that predicted targets of miR-484 were enriched in the protein co-expression module involved in synaptic transmission and regulation of long-term synaptic plasticity. Lastly, we performed a biological replication of our findings using small RNA sequencing technology and replicated the associations between miR-484 and (i) late-life depressive symptoms and (ii) clinical diagnosis of AD in an independent cohort. Together, these findings point to a molecular link between late-life depression and elevated risk for AD dementia through miR-484 for further mechanistic studies.

## Results

### Religious Orders Study (ROS)/Rush Memory and Aging Project (MAP) participants

A total of 300 non-demented ROS/MAP subjects were included in the global brain miRNA analysis of depressive symptoms assessed longitudinally. Table 1 lists the characteristics of the subjects. Briefly, these participants had an average of 16 years of education, 61% were women, 56% had normal cognition, and 44% had MCI (Table 1). These participants were followed for a mean of 7 years and a median of 6 years. Mean age at enrollment was 65 years and mean age at death was 87 years. The depression score for each individual averaged over all the follow-up years ranged from 0 to 7. On comparing ROS to MAP, there was no difference in the relative proportions of men and women in these two cohorts (*p* = 0.90). There was higher education level in ROS (mean years of education in ROS was 17.9 and in MAP 14.5; mean difference *p* < 2.2 × 10^−16^) and higher average depression score in MAP (average depression score over follow-up years in MAP was 1.46, range [0–7] and in ROS was it 1.12, range [0–6]; mean difference *p* = 0.03). We have adjusted for these differences by including sex and study (ROS vs. MAP) as the covariates in all analyses.

**Table 1:** Descriptive characteristics of the ROS/MAP participants (*N* = 300).

Characteristic	*N*	Percentage	
Sex (female)	183	61.0	
Diagnosis rendered at death			
Normal cognition	167	55.7	
Mild cognitive impairment	133	44.3	
Study			
ROS	164	54.7	
MAP	136	45.3	
	**Mean [SD]**	**Median**	**Range**
Age at enrollment	65.3 [7.2]	80.4	[65 to 98]
Age at death	86.9 [6.6]	86.9	[67 to 104]
Education	16.5 [3.5]	16.0	[5 to 26]
Depression score (average over all follow-up years)^a^	1.3 [1.3]	0.9	[0 to 7]
Rate of cognitive decline^b^	0.04 [0.04]	0.04	[−0.10 to 0.14]
Number of follow-up years	6.9 [3.9]	6.0	[1 to 17]
Postmortem interval (PMI)	7.3 [5.0]	5.9	[1 to 32.6]
RNA integrity number (RIN)	7.2 [1.0]	7.4	[5 to 9.9]
Global AD pathology	0.5 [0.5]	0.3	[0 to 2.3]

^a^CES-D has a possible score of 0 to 10 with the higher score, the more depressive symptoms

^b^Rate of cognitive decline refers to the slope of a person's cognitive trajectory over the follow-up years. Hence, the more negative the slope, the faster the decline

### Global miRNA association study of late-life depressive symptoms

**Fig. 1:**
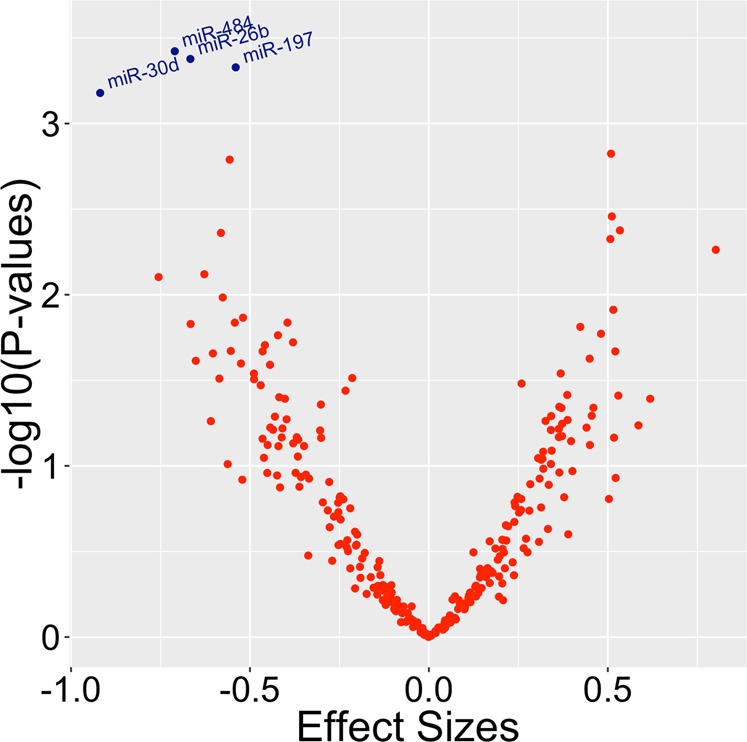
Volcano plot for global miRNA analysis of late-life depression assessed longitudinally. Four miRNAs were significantly associated with late-life depression after adjusting for sex; age at visit; global AD pathology; proportions of neurons, oligodendrocytes, astrocytes, and microglia; PMI; RIN; study; and batch at adjusted *p* < 0.05. These are miR-484, miR-26b-5p, miR-30d-5p, and miR-197-3p.

We found four miRNAs significantly associated with late-life depressive symptoms at an adjusted *p* value < 0.05 after accounting for sex, age, global AD pathology, cell type, postmortem interval (PMI), RNA integrity number (RIN), and study (Fig. 1, Table 2a, Supplementary Table 1). All four miRNAs (miR-484, miR-26b-5p, miR-30d-5p, and miR-197-3p) were less abundant in participants having greater depressive symptoms compared to participants with fewer depressive symptoms for a particular follow-up year (Table 2a, Supplementary Fig. 1). When we adjusted for the time interval between the time of death and the last clinical assessment for each subject, in addition to the covariates mentioned above, we found the same four miRNAs differentially expressed in late-life depression. Furthermore, to determine whether these results depended on AD pathology found at the end of life, a separate model that did not adjust for AD pathology was used, which showed similar findings except that miR-30d-5p had adjusted *p* value of 0.06 (instead of adjusted *p* value of 0.048). Together, these findings suggest that these miRNAs are associated with depressive symptoms independent of AD pathology.

**Table 2:** (A) Global miRNA association study of late-life depression (*N* = 300) and (B) depression-associated miRNAs are associated with dementia features.

miRNA	Coefficient	Coefficient SE	*p*	Adjusted *p*	Direction
(A)					
miR-484	−0.710	0.200	0.00038	0.04573	Lower miR-484 ~ greater depression
miR-26b-5p	−0.667	0.189	0.00042	0.04573	Lower miR-26b ~ greater depression
miR-197-3p	−0.540	0.154	0.00047	0.04573	Lower miR-197 ~ greater depression
miR-30d-5p	−0.919	0.270	0.00066	0.04841	Lower miR-30d ~ greater depression
**Predictor**	**Coefficient**	**Coefficient SE**	***p***	**Outcome**	**Direction**
(B)					
Regression model for rate of cognitive decline adjusting for relevant covariates^a^ (*N* = 482)					
miR-484	0.03	0.01	0.012	Cognitive decline	Lower miR-484 ~ faster decline
miR-197-3p	0.02	0.01	0.020	Cognitive decline	Lower miR-197 ~ faster decline
Regression model for clinical diagnosis of dementia adjusting for relevant covariates^a^ (*N* = 505)					
miR-484	−0.15	0.06	0.009	Dementia	Lower miR-484 ~ dementia

(A) miRNAs significantly associated with late-life depression at FDR *p* < 0.05 from a global miRNA association study of longitudinally assessed depression, adjusting for sex; age at visit; global AD pathology; proportions of neurons, oligodendrocytes, astrocytes, and microglia; postmortem interval; RIN; and study. All miRNAs were downregulated in greater depression. (B) Depression-associated miRNAs are also associated with dementia features after adjusting for sex; age at death; PMI; RIN; proportions of neurons, oligodendrocytes, astrocytes, and microglia; postmortem interval; RIN; and study

^a^Adjusted for sex, age at death, education, PMI, RIN, cell type, and study

### Depressive symptoms-associated miRNAs vs. dementia features

Given the observation that late-life depression is associated with increased incidence of dementia in several epidemiological studies,^1,2^ we examined associations between these four depressive symptom-associated miRNAs and the features of dementia to test whether these miRNAs share a common molecular mechanism with dementia. Interestingly, two miRNAs (miR-484 and miR-197-3p) were associated with the rate of cognitive decline over time after adjusting for sex; age at death; education; PMI; RIN; proportions of neurons, oligodendrocytes, astrocytes, and microglia; and study (miR-484, *β* = 0.03, *p* = 0.012, Supplementary Fig. 2a, Table 2b; miR-197-3p, *β* = 0.02, *p* = 0.020, Supplementary Fig. 2b, Table 2b, *N* = 482). Both miR-197-3p and miR-484 were downregulated in individuals with faster cognitive decline, as well as with greater depressive symptoms, consistent with the known detrimental effects of depression on cognitive aging.

Regarding the clinical diagnosis of dementia, miR-484 was significantly associated with clinical diagnosis of MCI or AD after adjusting for sex, age at death, education, PMI, RIN, cell-type proportions, and study (*β* = −0.15, *p* = 0.009, *N* = 505, Supplementary Fig. 2c, Table 2c). Lower miR-484 level was associated with higher odds for MCI or AD, as well as with greater depressive symptoms, which is consistent with the known negative effects of depression on dementia risk.

With regard to dementia pathologies, we examined global AD pathology, gross infarcts, microscopic infarcts, TDP-43, hippocampal sclerosis, cerebral amyloid angiopathy (CAA), atherosclerosis, and Lewy bodies in relation to these four miRNAs. However, we did not see a significant association between any of the four depressive symptom-associated miRNAs and any of the dementia pathologies. These findings are consistent with the observation that depressive symptoms are not directly associated with dementia pathologies.^3–6^

Since cognitive decline can be due to neurodegenerative pathologies or independent of them, we examined the associations between miR-484 and miR-197-3p and cognitive decline adjusting for all the measured neurodegenerative pathologies (i.e., global AD pathology, gross infarcts, microscopic infarcts, TDP-43, hippocampal sclerosis, CAA, atherosclerosis, and Lewy bodies) and sex, age at death, PMI, RIN, study, and cell-type proportions. Using this model, we found that only miR-197-3p was significantly associated with cognitive decline (miR-197-3p, *β* = 0.023, *p* = 0.016, *N* = 390). This suggests that miR-197-3p contributes to cognitive decline via mechanisms independent of the known dementia pathologies and may be a target for further studies of cognitive resilience.

### Enrichment of targets of miR-484 and miR-197-3p in protein co-expression modules

**Fig. 2:**
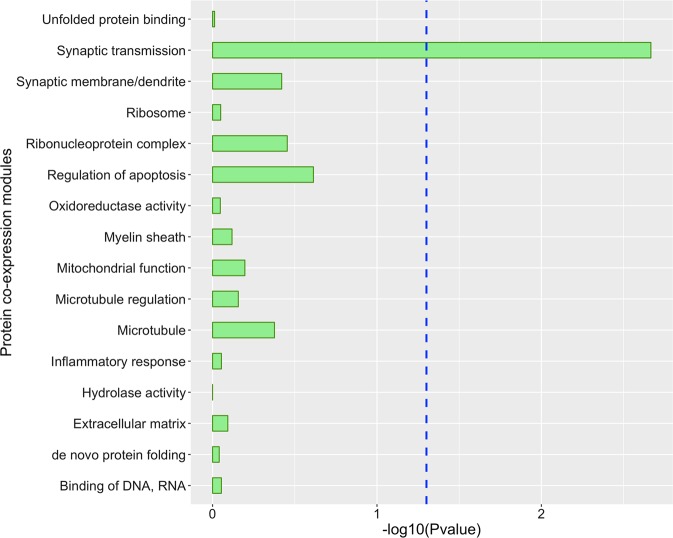
Enrichment of the predicted targets of miR-484 in BLSA protein co-expression modules. The *y*-axis lists the 16 brain protein co-expression modules and their enriched biological activities from Seyfried et al.^14^ The blue line perpendicular to the *x*-axis represents Benjamini–Hochberg adjusted *p* < 0.05.

Since a miRNA represses expression of its target genes by either destabilizing their transcripts or repressing the translation of the transcripts to proteins, the effects of miRNAs are likely best observed at the protein level. Hence, we asked whether the targets of miR-484 or miR-197-3p would be enriched in any of the 16 published protein co-expression modules derived from the proteomes of the Baltimore Longitudinal Study of Aging (BLSA) cohort.^14^ We found that targets of miR-197-3p were not enriched in any of these modules. Interestingly, the targets of miR-484 were enriched in the M1 turquoise module, which is involved in synaptic transmission and regulation of long-term neuronal synaptic plasticity (enrichment *p* = 0.002; Benjamini–Hochberg (BH) adjusted *p* = 0.034; Fig. 2). There were 46 genes that were common between the predicted targets of miR-484 and module M1 turquoise (Supplementary Table 2). Of note, since the targets of miR-484 and miR-197 were predicted by TargetScan, which may include false positives, we filtered each target by the cumulative weighted context score (CWCS) to reduce potential false positives.^15^ The CWCS is an estimate of how effective the miRNA binds to and represses the expression of that gene. The more negative the CWCS, the more effective the miRNA can suppress the expression of that target.^15^ Filtering targets of miR-484 and miR-197 to include only targets with CWCS < −0.1 for this enrichment analysis led to recapitulating our initial results but with a greater degree of significance, i.e., targets of miR-484 were enriched in the M1 turquoise module involved in synaptic transmission and regulation of synaptic plasticity (enrichment *p* = 0.0006; BH adjusted *p* = 0.010; Supplementary Fig. 3).

### Biological replication of associations between miR-484 and depressive symptoms and AD

An independent set of 160 ROS/MAP subjects was available as a biological replication cohort and their characteristics are presented in Table 3. In this replication cohort, we found that lower miR-484 level in the dorsolateral prefrontal cortex (dlPFC) was significantly associated with greater depressive symptoms longitudinally assessed after adjusting for sex, age at visit, RIN, PMI, sequencing batch, global AD pathology burden, and study (miR-484, *β* = −1.04 [0.46], *p* = 0.024, *N* = 160; Supplementary Fig. 4). We did not have transcriptomic data to estimate cell-type composition to adjust for in this biological replication analysis. This finding is consistent with the inverse association between miR-484 and depressive symptoms found in the discovery cohort. We did not find an association between dlPFC miR-197-3p and depressive symptoms (*β* = 0.03 [0.58], *p* = 0.96).

**Table 3:** Descriptive characteristics of the ROS/MAP biological replication cohort (*N* = 160).

Characteristic	*N*	Percentage	
Sex (female)	111	69.4	
Diagnosis rendered at death			
Normal cognition	50	31.3	
Mild cognitive impairment	50	31.2	
Alzheimer dementia	60	37.5	
Study			
ROS	18	11.3	
MAP	142	88.7	
	**Mean [SD]**	**Median**	**Range**
Age at enrollment	82.0 [6.4]	81.9	[66–97]
Age at death	90.3 [6.2]	90.3	[71–104]
Education	14.9 [3.2]	15.0	[4–25]
Depression score (average over all follow-up years)^a^	1.4 [1.4]	1.0	[0–8]
Number of follow-up years	6.3 [4.4]	6.0	[0–19]
Postmortem interval (PMI)	9.2 [5.7]	7.3	[2.3–38.3]
RNA integrity number (RIN)	5.4 [1.5]	5.4	[2.1–8.1]

^a^CES-D has a possible score range of 0 to 10; higher scores indicate greater depressive symptoms

Furthermore, we found that lower expression of miR-484 was associated with MCI or AD in this biological replication cohort after adjusting for sex, age at death, PMI, RIN, and education, consistent with what we found in the discovery cohort (*β* = −2.35 [0.86]; *p* = 0.0060; *N* = 160).

## Discussion

Our primary objective was to investigate molecular links between late-life depression and increased AD and vascular dementia risk. To that end, we performed a global brain miRNA association study of late-life depressive symptoms that were assessed annually and longitudinally over a mean of 7 years in two community-based cohorts. We found four miRNAs significantly downregulated in greater depressive symptoms (miR-484, miR-197-3p, miR-26b-5p, and miR-30d-5p). Among these depressive symptom-associated miRNAs, miR-484 and miR-197-3p were also associated with faster decline of cognition over time and miR-484 with higher probability for having a clinical diagnosis of MCI or AD dementia. These findings suggest that miR-484 and miR-197-3p may play a significant role in the association between late-life depression and elevated risk for AD and vascular dementia. Importantly, the association between miR-484 expression level and depressive symptoms was replicated in an independent ROS/MAP cohort of 160 participants through small RNA sequencing technology. This biological replication via a different miRNA profiling technology increases the confidence of the importance of miR-484 in late-life depression. Furthermore, we replicated the association between lower miR-484 expression and MCI or AD dementia in a biological replication cohort. Of note, our finding of lower miR-484 expression being associated with AD dementia is consistent with another study that found decreased level of temporal cortex miR-484 in AD compared with no cognitive impairment.^16^ Taken together, these findings highlight a molecular link between late-life depressive symptoms and elevated risk for AD dementia through miR-484.

miR-484 regulates mitochondrial fission;^17,18^ thus altered miR-484 expression can lead to altered mitochondrial fission. Mitochondrial fission and fusion refer to dynamic interactions among mitochondria to establish organelle shape, size, number, and allow for mixing of mitochondrial contents.^19^ From our findings, it can be inferred that depression may increase risk for AD dementia through altered mitochondrial fission. The connection between mitochondrial fission and AD dementia is consistent with prior studies showing that altered balance of mitochondrial fission and fusion is an important mechanism leading to neuronal dysfunction in AD dementia brain.^20,21^ Furthermore, mitochondria in neurons are crucial for maintaining synaptic function because they regulate synaptic transmission by generating ATP to power the process and by modulating presynaptic calcium level, which determines the release of neurotransmitters.^22–24^ Moreover, we found that the predicted targets of miR-484 were enriched in the protein co-expression module involving synaptic transmission and regulation of long-term neuronal synaptic plasticity. We hypothesize that miR-484 acts through its effects on synaptic transmission and neuronal synaptic plasticity in elevating risk for AD and vascular dementia, which should be tested in model systems.

Our finding that prefrontal cortex miR-484 was down-regulated in individuals with greater depressive symptoms is consistent with results from two prior studies of bipolar disorder, a syndrome predominated by severe depressive symptoms, that observed downregulation of prefrontal cortex miR-484 in bipolar disorder.^25^ Bipolar disorder and depression share at least two features. First, individuals with bipolar disorder are depressed the majority (70–81%) of their ill-time.^26^ Second, there is a significant shared heritability between depressive symptoms and bipolar disorder (*r* = 0.35).^27^ Our finding of the connection between late-life depression and AD dementia through miR-484 is complementary to the finding that polygenic risk score (PRS) of major depression predicted the conversion of non-depressed amnestic MCI to AD dementia.^28^ The effect of the PRS of major depression on the conversion of MCI to AD was mediated through left hippocampal volume.^28^ Consistently, another study found different patterns of hippocampal atrophy among participants with AD, amnestic MCI, and late-life depression.^29^

In a prior study, miR-135 was found to be lower in the brain of 11 depressed suicide human subjects by Issler and colleagues.^30^ miR-135, however, was not associated with depressive symptoms in the ROS/MAP cohorts (*p* = 0.06). This is likely due to two reasons. First, Issler and colleagues examined the brainstem and not the dlPFC. Furthermore, Issler and colleagues examined miR-135 in five different subnuclei of the raphe. Differential expression of miR-135 in depression was only detected in two subnuclei, the dorsal raphe and raphe magnus, reflecting the brain region-specific expression of this miR-135. Second, the association between miR-135 and depression was found by Issler and colleagues in depressed suicide victims, which typically reflects severe major depression, while we studied late-life depressive symptoms in a community-based cohort with mild depressive symptoms; very few of our participants had major depression. Thus it is plausible that the severity of depression may influence the abundance of miR-135. In addition, miR-132 was found to be associated with AD and pathologies by Lau and colleagues and also by our group.^31,32^ However, we did not find an association between miR-132 and depressive symptoms here (*p* = 0.39).

Depression and AD dementia are relatively common conditions in older age and are frequently comorbid.^33,34^ Both depression and AD dementia are heterogeneous etiologically and clinically.^33^ Furthermore, the question of whether depression is a risk factor for dementia or part of its prodrome has not been resolved. In other word, the neurodegenerative process of developing AD dementia may contribute to the development of late-life depression or vice versa.^33^ Hence, we here examined brain miRNA signature of depressive symptoms assessed longitudinally in subjects with either normal cognition or MCI to identify the molecular basis behind the association between late-life depression and higher risk for AD dementia. One limitation of our study is that it has miRNA profile from only one region of the brain, the dlPFC. Examining miRNA profiles from different regions of the brain would provide a more comprehensive picture of the association between late-life depression and brain miRNA changes. However, the dPFC is a region that is relatively less affected in AD and thus more likely to show more subtle changes compared to areas where pathologies occur early (e.g., hippocampus or temporal cortex). Another limitation of the study is that this is a postmortem brain study, therefore it is cross-sectional by nature and no causal relationship can be established.

Our study has several strengths. First, it is the first global brain miRNA association study of late-life depressive symptoms to the best of our knowledge. Second, in our study, late-life depressive symptoms were assessed annually and longitudinally over a mean of 7 follow-up years. Third, this is the first study to observe a link between late-life depressive symptoms and dementia at the molecular level, via miRNAs. Fourth, we rigorously controlled for potential confounding factors in our analyses including postmortem brain interval and tissue heterogeneity. Fifth, we performed a biological validation of our initial findings using next-generation small RNA sequencing in a separately ascertained collection of samples.

In conclusion, we show that late-life depressive symptoms are linked to the downregulation of prefrontal cortex miR-484. Furthermore, the association between late-life depression and elevated risk for dementia observed in 23 prospective epidemiological studies^1,2,35^ may be partially explained by miR-484 through its effects on synaptic transmission, synaptic plasticity, and mitochondrial fission. Further mechanistic studies are needed to confirm these observations.

## Methods

### Participants

Participants were from two longitudinal clinical–pathologic cohort studies of aging and AD—ROS and MAP.^36^ ROS is comprised of older Catholic priests, nuns, and monks throughout the USA. MAP recruits older lay persons from the greater Chicago area. Both studies involve detailed annual cognitive and clinical evaluations and brain autopsy. Participants provided informed consent, signed an Anatomic Gift Act, and a repository consent to allow their data and biospecimens to be repurposed. The studies were approved by the Institutional Review Board of Rush University Medical Center. To be included in our global miRNA association study of depressive symptoms, participants must not carry a final clinical diagnosis of dementia.

### Neuropsychiatric phenotypes

Depressive symptoms were assessed annually using the 10-item version of the Center for Epidemiological Studies Depression scale (CES-D).^37^ The CES-D captures the major symptoms of depression as identified in clinical and factor analytical studies, i.e., depressed affect, positive affect, somatic complaints, and interpersonal problems, with a reliability of 0.8.^37^ The possible score range for the CES-D is 0–10, with higher score indicating more depressive symptoms.

Final clinical diagnosis of AD dementia is determined at the time of death by a neurologist with expertise in dementia using all available clinical data but blinded to postmortem data. The diagnosis was based on the recommendation of the National Institute of Neurological and Communicative Disorders and Stroke and the AD and Related Disorders Association.^38^ Case conferences including one or more neurologists and a neuropsychologist were used for consensus as necessary*.* Clinical diagnoses of cognitive status can include no cognitive impairment, MCI, or AD.

Rate of decline of cognitive performance is the individual rate of decline of global cognitive performance over time. Annually, 21 cognitive tests were administered to each ROS/MAP participant with 19 in common. The raw scores from 19 cognitive tests were standardized to a *Z* score with respect to the mean and standard deviation of the cohort at the baseline visit. These *Z* scores were averaged to create the composite annual global cognitive score. Rate of cognitive change is the random slope with respect to follow-up years in the mixed linear model in which the annual global cognitive performance is the longitudinal outcome; follow-up year is the independent variable with random effect per subject; and age at recruitment, sex, and years of education are the covariates.

#### Dementia pathologies

Brain autopsy was performed by examiners who were unaware of deceased participants’ clinical information and have been described in detail before.^39,40^ Nine brain regions of interest (i.e., midfrontal, midtemporal, inferior parietal, anterior cingulate, entorhinal and hippocampal cortices, basal ganglia, thalamus, and midbrain) were dissected and stained for assessment of pathology. Global AD pathology (i.e., neuritic plaques, diffuse plaques, and neurofibrillary tangles) was visualized in five cortical regions using a modified Bielschowsky silver stain. Counts of silver-stained neuritic plaques, diffuse plaques, and neurofibrillary tangles were used to create a continuous measure of AD global pathology. The square root of this global pathology measure was used in our analyses to improve its normal distribution. Chronic gross infarcts were identified visually by examining slabs and pictures from both hemispheres and confirmed histologically and was treated as a dichotomous variable (present vs. absent) in our analyses. Microinfarcts were those that were not visible to the naked eye but were identified under microscope using hematoxylin and eosin stain in a minimum of nine regions, including six cortical regions, two subcortical regions, and midbrain. Microinfarcts were treated as present or absent in our analyses. Lewy body pathology was assessed using antibodies to α-synuclein in six regions, including substantia nigra, limbic, and neocortices, and treated as present or absent in our analyses. Hippocampal sclerosis was identified as severe neuronal loss and gliosis in hippocampus or subiculum using hematoxylin and eosin stain and treated as present or absent in analyses.^41^ TDP-43 cytoplasmic inclusions were assessed in six regions using antibodies to phosphorylated TDP-43. Inclusions in each region were rated on a six-point scale and the mean of the regional scores was created.^42^ TDP-43 scores were dichotomized into absent (i.e., mean score of 0) or present (mean score >0) in our analyses. Cerebral atherosclerosis was assessed by visual inspection of vessels in the circle of Willis and rated as absent, mild, moderate, or severe and treated as a semiquantitative variable in our analyses.^43^ CAA was assessed using amyloid-β immunostaining in four regions (i.e., midfrontal, inferior temporal, angular, and calcarine). In each region, meningeal and parenchymal vessels were assessed for amyloid deposition and scored from 0 to 4, where 0 indicates no deposition, 1 refers to scattered segmental but no circumferential deposition, 2 means circumferential deposition up to 10 vessels, 3 reflects circumferential deposition in >10 vessels and up to 75% of the vessels, and 4 indicates circumferential deposition in >75% of the vessels.^44^ The score for each region was the maximum of the meningeal and parenchymal scores, and a continuous summary score was created by averaging scores across the regions.^44^

### miRNA quantification and quality control

Quantification and quality control of miRNA profiles were described in detail before.^32^ Briefly, total RNA was extracted from the full-thickness cortex with dissection of the underlying white matter of the dlPFC in the Brodmann areas 9 and 46 (BA 9/46). Then small RNA was isolated from total RNA and profiled using the Nanostring platform. RIN ranged from 5 to 10. Samples were randomized with respect to clinical diagnosis of cognitive status to minimize batch effects. miRNAs from the Nanostring RCC files were re-annotated using miRBase v19. After correcting for the probe-specific backgrounds, a three-step filtering of miRNA expression levels was performed. First, miRNA with missing expression levels in >95% of samples were removed. Second, samples with >95% of miRNAs with missing expression were removed. Third, all miRNA whose absolute values were <15 in at least 50% of the samples were removed to eliminate miRNA that had negligible expression in brain samples. After the miRNA and sample filtering, the dataset consisted of 292 miRNAs. Combat was used to remove batch effects and quantile normalization was performed.^45^ All miRNA data are available at https://www.synapse.org/#!Synapse/syn3219045.

### Estimate of brain cell-type proportions

The total RNA extracted from postmortem brain dlPFC in ROS/MAP subjects was used for miRNA profiling as well as for RNA sequencing as described in detail before.^46^ Hence, we used the transcriptomes derived from RNA sequencing for these subjects to estimate the proportions of neurons, astrocytes, oligodendrocytes, and microglia for the dlPFC brain tissue from which we profiled miRNAs. We used the proportions of these four cell types as covariates in all our miRNA analyses to control for the heterogeneity of cell types in brain tissues. We used CIBERSORT pipeline^47^ and Darmanis’ single-cell RNA sequencing expression profiles from human brain tissue samples as the reference ^48^ to estimate brain cell-type composition.

### Global miRNA association study of depressive symptoms

Using linear mixed model, we examined the association between longitudinally assessed depressive symptoms and global miRNA profile, adjusting for sex; age at visit; global AD pathology burden; proportions of neurons, oligodendrocytes, astrocytes, and microglia; PMI; RIN; and study (ROS or MAP). Specifically, depression score was modeled as the outcome and miRNA as a predictor, with a random intercept and random slope for each subject with respect to the follow-up year, adjusting for the above-mentioned covariates. In this linear mixed model, we tested whether the miRNA abundance had a non-zero effect on the depression scores. By using a mixed effect on follow-up year (a covariate in this model), we accounted for the individual-specific change in depression score from year to year in the association test. To address multiple testing, we used BH method to control the false discovery rate and declared significantly associated miRNAs as those with an adjusted *p* < 0.05.^49^

### Depressive symptom-associated miRNAs vs. dementia features

We examined associations between depressive symptom-associated miRNAs from the global miRNA analysis above and the dementia-related features. These dementia features include (i) rate of decline of cognitive performance; (ii) clinical diagnosis of dementia; and (iii) neurodegenerative pathologies, including global AD pathology, Lewy bodies, TDP-43, cerebral atherosclerosis, gross infarcts, chronic microinfarcts, CAA, and hippocampal sclerosis. We included all ROS/MAP participants with miRNA profiles in this analysis, hence the sample size for these analyses is >300. We used general linear regression model in which specific dementia feature was the outcome, miRNA was the predictor, and sex; age at death; education; PMI; RIN; proportions of neurons, oligodendrocytes, astrocytes, and microglia; and study are the covariates.

### Target genes of miR-484 and miR-197 and protein co-expressed modules

A published study identified 16 modules of co-expression proteins in the BLSA.^14^ Since an miRNA represses the expression of its target genes by either destabilizing the transcripts or repressing the translation of transcripts to proteins, the effects of miRNAs ought to be most apparent at the protein level. Hence, we asked whether the targets of miR-484 or miR-197 would be enriched in any of these protein co-expression modules. To that end, predicted targets of miR-484 and miR-197 were identified from TargetScan database v7.2.^15^ Next, we asked whether these targets were enriched in any of the 16 modules of co-expressed proteins mentioned above using gene set enrichment analysis and adjusting for multiple testing with BH method.

### Biological replication cohort

For a biological replication cohort, we used postmortem brain samples from the dlPFC BA 9/46 of 160 independent ROS/MAP subjects who were not part of the discovery cohort. Total RNA was extracted from the cortically dissected dlPFC with TRIzol (Invitrogen, Carlsbad, CA, USA) following the manufacturer’s protocol. Total RNA was used to create small RNA libraries using New England Biolabs’ NEBNext® Multiplex Small RNA Library Prep kit following the manufacturer’s protocol. Small RNA libraries were sequenced single-end read on the Illumina HiSeq3000. To avoid batch effects, samples were prepared for sequencing in batches of 8 that were randomized based on sex and cognitive diagnosis for RNA extraction, and by sex, cognitive diagnosis, RIN, PMI, pathologies, depression score, and study for small RNA library preparation and sequencing.

Quality of the sequencing data was inspected with FastQC. Adapters were trimmed with Trimmomatic.^50^ Trimmed reads were aligned to the miRBase v.22^51^ with SHRIMP aligner.^52^ Counts of aligned miRNAs were normalized with variance stabilization transformation, which performs log_2_ transformation, normalizes for library size, and transforms the counts to approximately homoscedastic.^53^ After variance stabilization transformation, miR-484 and miR-197-3p were extracted for association analysis with longitudinal depressive symptoms using linear mixed model adjusting for the same covariates as was done in the discovery dataset.

### Reporting summary

Further information on research design is available in the Nature Research Reporting Summary linked to this article.

## Supplementary information

Supplementary Information

Reporting Summary

## Data Availability

All miRNA data are available at https://www.synapse.org/#!Synapse/syn3219045. ROS/MAP Clinical and Pathologic Data are available at http://www.radc.rush.edu. Proteomic data from the Baltimore Longitudinal Study on Aging (BLSA) study are available at https://www.synapse.org/#!Synapse:syn3606086.
