# Supplementary material for: Brain microRNAs associated with late-life depressive symptoms are also associated with cognitive trajectory and dementia
Source: NPJ Genom Med. 2020 Feb 6;5:6. doi: 10.1038/s41525-019-0113-8 (PMC7004995; doi:10.1038/s41525-019-0113-8)
Supplement: Supplementary file 1 — Supplementary Information [file 41525_2019_113_MOESM1_ESM.pdf]

Supplementary Figure 1: All four depression-associated miRNAs showed lower abundance in participants with greater depressive symptoms. The blue line represents the best fit regression line and the shaded grey area represents the 95% confidence interval for the regression.

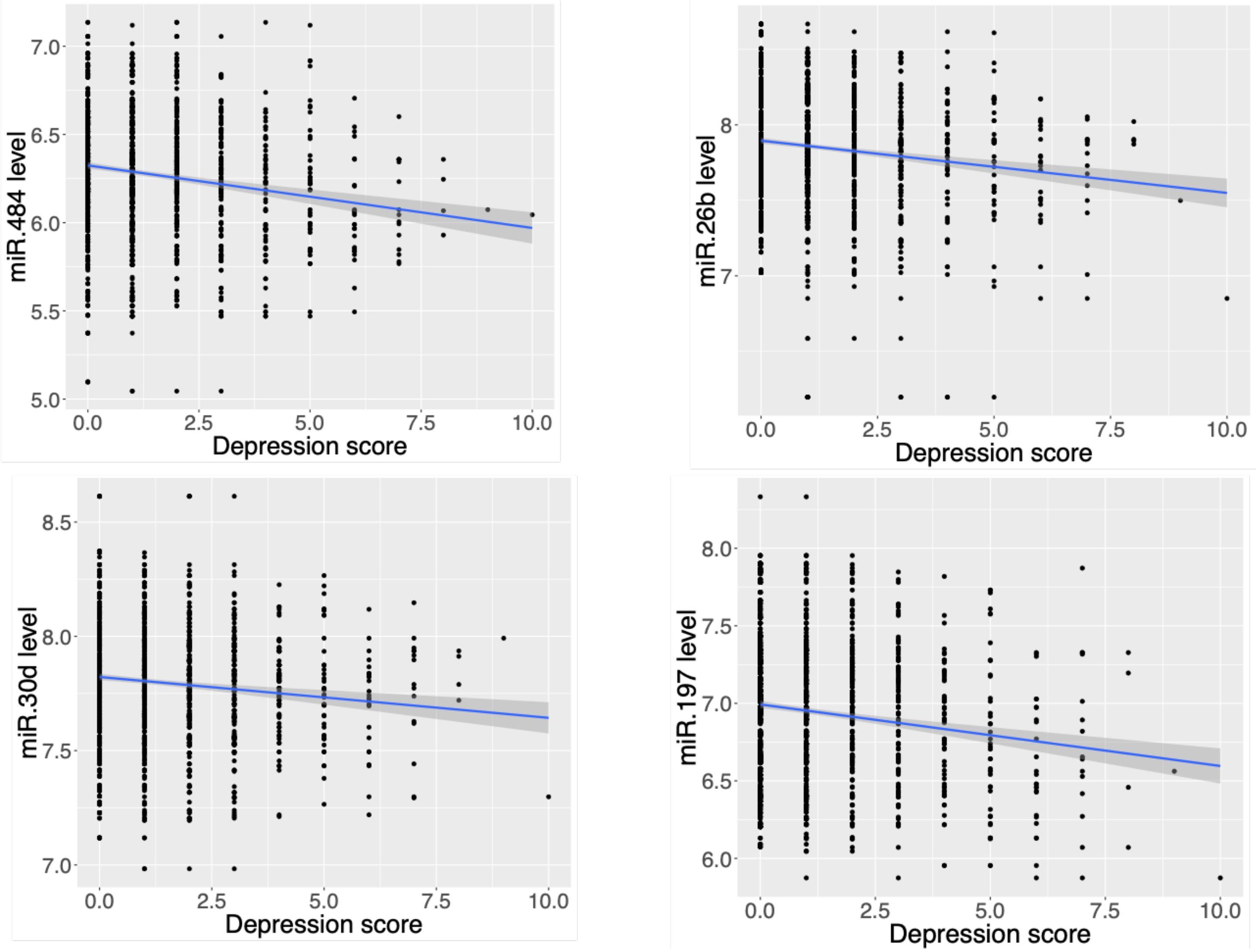

Supplementary Figure 2: Depression-associated miRNAs were associated with dementia features. **A)** miR-484 was significantly down-regulated in faster cognitive decline (p=0.012; N=482). **B)** miR-197 was significantly down-regulated in faster cognitive decline (p=0.020; N=482). **C)** down-regulation of miR-484 was associated with higher probability for a diagnosis of MCI or AD (p=0.009, N=505).

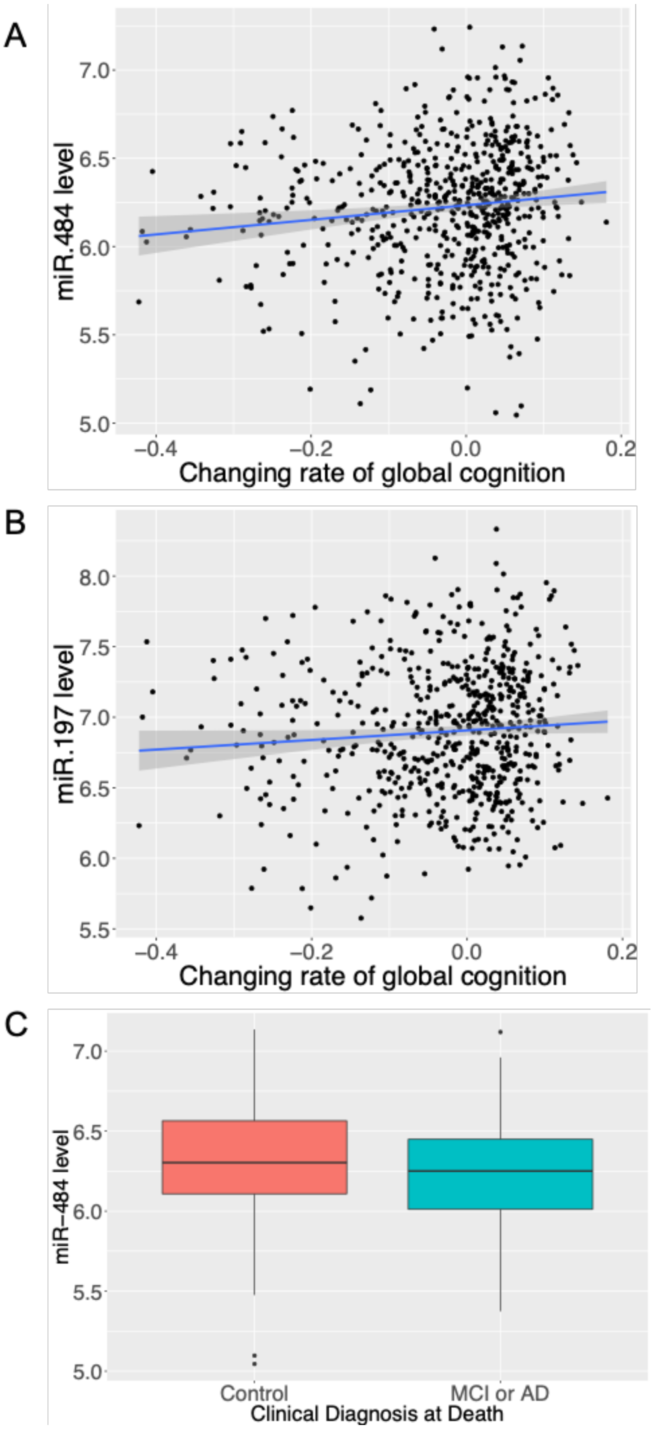

Supplementary Figure 3: Enrichment of the predicted targets of miR-484 in BLSA protein co-expression modules. The potential targets of miR-484 were those with cumulative weighted context score < -0.1. The y-axis lists the 16 brain protein co-expression modules and their enriched biological activities from Seyfrieds et al 2017. The blue line perpendicular to the x-axis represents Benjamini-Hochberg adjusted p <0.05.

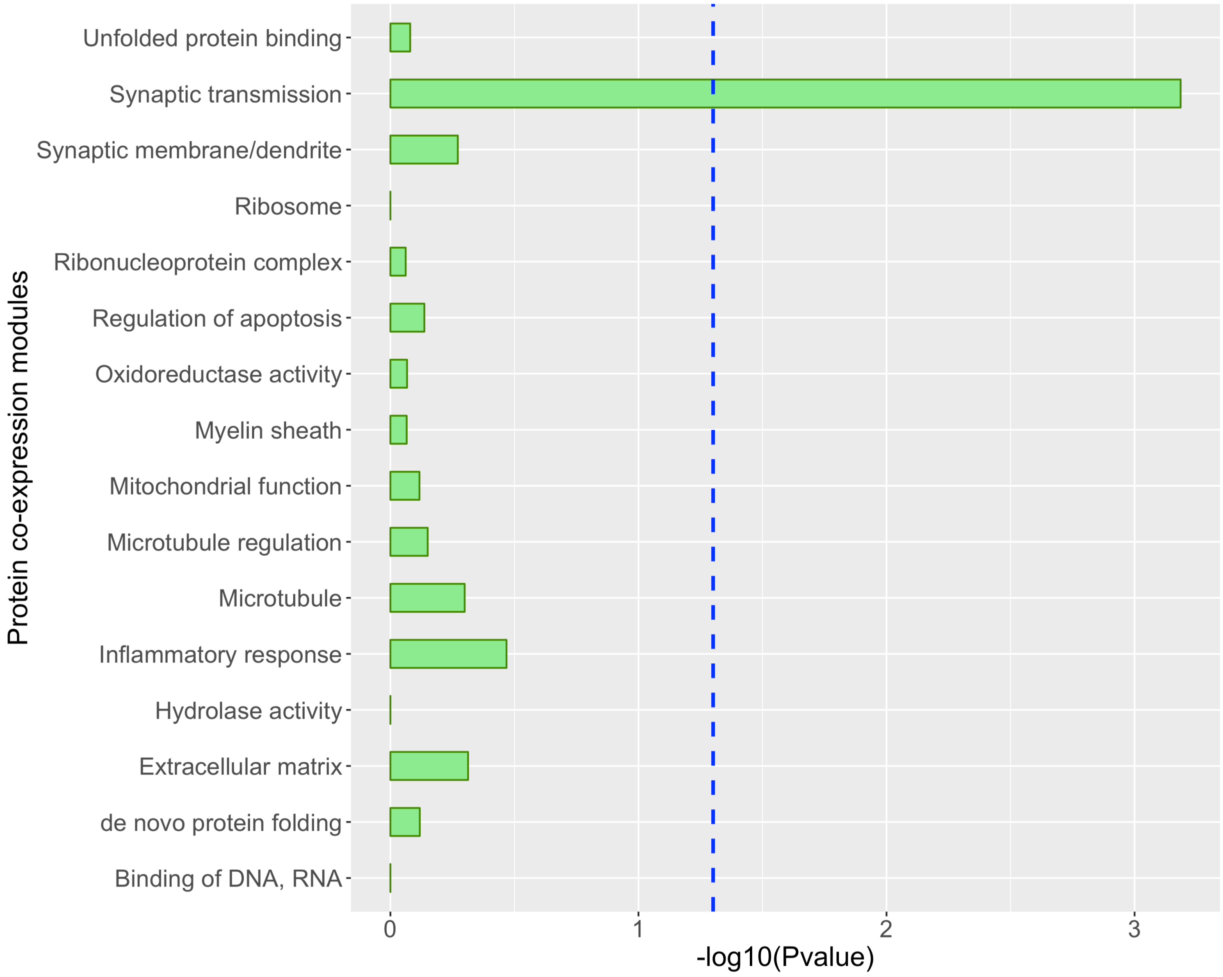

Supplementary Figure 4: In our biological replication cohort, miR-484 was inversely associated with the level of depressive symptom severity after adjusting for sex, age at visit, RIN, PMI, global AD pathology burden, study, and sequencing batch (N=160).

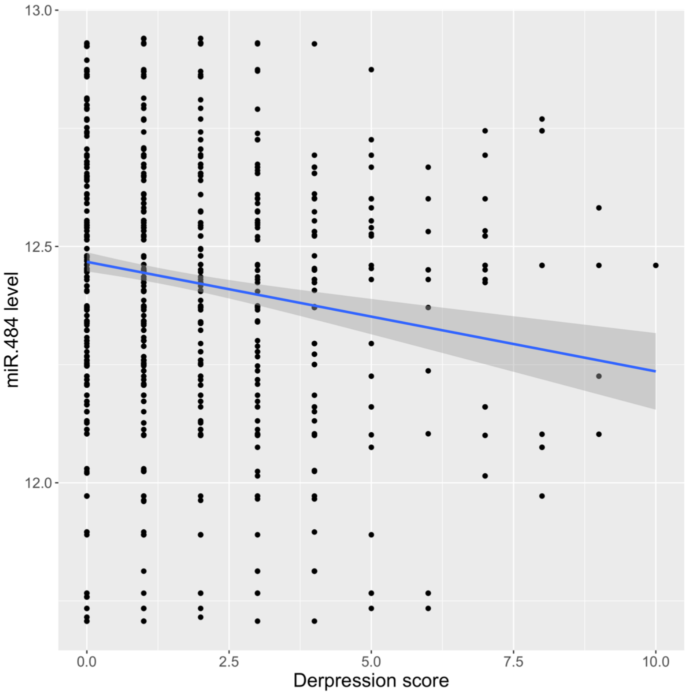

Supplementary Table 1: Global miRNA association study of late-life depressive symptoms adjusting for sex, age, global AD pathologies, PMI, RIN, proportions of neurons, astrocytes, oligodendrocytes, and microglia, and study

miRNA	beta	SE_beta	*p*	Adjusted *p*
hsa.miR.484	-0.7125696	0.19969296	0.000359269	0.04640937
hsa.miR.26b	-0.6696429	0.18891811	0.00039318	0.04640937
hsa.miR.197	-0.5393892	0.15439982	0.000476809	0.04640937
hsa.miR.30d	-0.9209513	0.26975993	0.000640245	0.0467379
hsa.miR.125b	0.51258953	0.16024153	0.001379742	0.08057693
hsa.miR.124	-0.555506	0.17668994	0.001666784	0.08111683
hsa.miR.300	0.51157984	0.17502453	0.003467838	0.13621322
hsa.miR.142.3p	0.53742911	0.18644256	0.003944764	0.13621322
hsa.miR.29b	-0.5834141	0.20378712	0.004198353	0.13621322
hsa.miR.1206	0.50625508	0.17951631	0.004800774	0.14018261
hsa.miR.142.5p	0.8033347	0.28852543	0.005364722	0.14240897
hsa.miR.23b	-0.7632721	0.28414697	0.007227247	0.16261557
hsa.miR.155	-0.6310626	0.23497915	0.007239734	0.16261557
hsa.miR.362.5p	-0.5822941	0.2246541	0.009543124	0.19904229
hsa.miR.770.5p	0.51465131	0.20573109	0.012364299	0.22141786
hsa.miR.487b	-0.5202978	0.21049405	0.013443729	0.22141786
hsa.miR.20a_hsa.miR.20b	-0.671497	0.27323373	0.013987209	0.22141786
hsa.miR.196a	-0.3959787	0.16173325	0.014351427	0.22141786
hsa.miR.222	-0.5412123	0.22198275	0.01476539	0.22141786
hsa.miR.548b.3p	0.42173925	0.17466507	0.015754241	0.22141786
hsa.miR.220a	0.48323311	0.20128243	0.016360551	0.22141786
hsa.miR.128	-0.4196713	0.17723385	0.017889579	0.22141786
hsa.miR.410	-0.3810636	0.16178649	0.018505336	0.22141786
hsa.miR.491.3p	-0.4609165	0.19650252	0.018996426	0.22141786
hsa.miR.150	-0.5596451	0.24035156	0.019888543	0.22141786
hsa.miR.335	0.52482858	0.22638622	0.020433642	0.22141786
hsa.miR.885.5p	-0.4660793	0.2019971	0.021034964	0.22141786
hsa.miR.30b	-0.6037881	0.26360251	0.021990917	0.22141786
hsa.miR.1248	0.4523776	0.19847915	0.022654005	0.22141786
hsa.miR.129.5p	-0.6553795	0.28927213	0.023474915	0.22141786
hsa.miR.133a	-0.5312906	0.23455526	0.023506691	0.22141786
hsa.miR.30c	-0.4442528	0.19908246	0.025647934	0.2340374
hsa.miR.543	-0.4924607	0.22373699	0.027731084	0.24078317
hsa.let.7b	-0.5935636	0.27115063	0.028592843	0.24078317
hsa.miR.891b	0.3662935	0.16858476	0.029798708	0.24078317
hsa.miR.633	-0.2146288	0.0991628	0.030433033	0.24078317
hsa.miR.26a	-0.4905832	0.22676457	0.030510196	0.24078317
hsa.miR.7	0.25938475	0.12150355	0.032778044	0.25187339
hsa.miR.1246	-0.2348418	0.11132404	0.034898569	0.25807506
hsa.miR.138	-0.465706	0.22144736	0.0354649	0.25807506
hsa.miR.758	0.3910081	0.1867044	0.036236567	0.25807506
hsa.miR.203	-0.4219578	0.20334907	0.037982662	0.25855165
hsa.let.7g	0.53147162	0.25624785	0.038074387	0.25855165

hsa.miR.769.3p	-0.4028525	0.19643354	0.040283176	0.2673338
hsa.miR.324.3p	0.61016936	0.30179511	0.043197011	0.27294504
hsa.miR.218	0.37247234	0.18546144	0.044605625	0.27294504
hsa.miR.19b	0.46024967	0.23012655	0.045501869	0.27294504
hsa.miR.216a	-0.2983661	0.14970031	0.046251655	0.27294504
hsa.miR.639	0.36139202	0.18194468	0.04700289	0.27294504
hsa.miR.15b	0.45823108	0.23304761	0.049269139	0.27294504
hsa.miR.369.3p	-0.4300076	0.22103348	0.051721935	0.27294504
hsa.miR.518e	0.3292501	0.16959392	0.05220944	0.27294504
hsa.miR.640	0.38972595	0.20109091	0.052616081	0.27294504
hsa.miR.769.5p	-0.398012	0.205947	0.053286189	0.27294504
hsa.miR.566	0.3369957	0.17519888	0.054416809	0.27294504
hsa.miR.148b	0.59351438	0.3087599	0.054574042	0.27294504
hsa.miR.383	-0.6093182	0.31726834	0.054793055	0.27294504
hsa.miR.183	0.37475065	0.19545727	0.055199552	0.27294504
hsa.miR.485.5p	0.44528445	0.23390607	0.05695068	0.27294504
hsa.miR.299.5p	0.3664073	0.19352697	0.058316134	0.27294504
hsa.miR.433	-0.4121804	0.21793902	0.058588974	0.27294504
hsa.miR.361.5p	-0.4447994	0.23552214	0.05894986	0.27294504
hsa.let.7f	-0.434877	0.23294109	0.061916005	0.27294504
hsa.miR.548d.5p	0.3395683	0.18258769	0.062920256	0.27294504
hsa.miR.1308	-0.3026138	0.16296884	0.063328064	0.27294504
hsa.miR.191	0.37509007	0.20312884	0.064810591	0.27294504
hsa.miR.135b	0.36689963	0.19879139	0.064942824	0.27294504
hsa.miR.374a	-0.3032139	0.1653721	0.066724555	0.27294504
hsa.miR.1283	-0.468501	0.2560101	0.067248463	0.27294504
hsa.miR.15a	0.51942954	0.28384115	0.067249816	0.27294504
hsa.miR.376c	-0.4125938	0.22550243	0.067300088	0.27294504
hsa.miR.125a.3p	-0.3702436	0.20235703	0.067301516	0.27294504
hsa.miR.574.3p	-0.3676832	0.20164673	0.068242384	0.27296954
hsa.miR.92b	-0.3820533	0.212098	0.071654709	0.27816775
hsa.miR.30e	-0.455355	0.25345629	0.072402003	0.27816775
hsa.miR.154	0.45354614	0.2531226	0.073164331	0.27816775
hsa.miR.200b	0.39488994	0.22053111	0.073352454	0.27816775
hsa.miR.296.5p	-0.4193369	0.23755167	0.077522619	0.29021288
hsa.miR.423.3p	-0.3438241	0.19701977	0.08096314	0.29597164
hsa.miR.1912	0.34347448	0.19690041	0.08108812	0.29597164
hsa.miR.548g	0.31725476	0.18394297	0.084573395	0.3019917
hsa.miR.378	-0.3700934	0.21473896	0.084805887	0.3019917
hsa.miR.193a.3p	0.32058355	0.18769362	0.08763352	0.3080655
hsa.miR.125a.5p	-0.4613423	0.27162877	0.089426706	0.3080655
hsa.miR.1266	0.30565063	0.18010107	0.089676602	0.3080655
hsa.miR.224	0.3116556	0.18625791	0.094278552	0.32010857
hsa.miR.651	0.34134026	0.20580522	0.097204543	0.32624973
hsa.miR.664	-0.5601008	0.33950741	0.09899501	0.32848344
hsa.miR.525.5p	0.32312655	0.19666821	0.100382218	0.32934391
hsa.miR.181b_hsa.miR.181d	-0.4613309	0.28239658	0.102337015	0.33202676

hsa.miR.421	0.39931736	0.24908081	0.10889847	0.34305444
hsa.miR.328	-0.373054	0.23294464	0.109272679	0.34305444
hsa.miR.1180	-0.4273023	0.26816288	0.111060799	0.34305444
hsa.let.7d	0.5306327	0.33326361	0.111332769	0.34305444
hsa.miR.499.5p	-0.3615538	0.22752554	0.112044763	0.34305444
hsa.miR.409.5p	-0.3413405	0.2163779	0.114675885	0.34305444
hsa.miR.455.3p	0.35857632	0.22764635	0.115222665	0.34305444
hsa.miR.192	-0.527609	0.33565961	0.115983684	0.34305444
hsa.miR.376a	-0.3370637	0.21462821	0.116309553	0.34305444
hsa.miR.889	-0.2819134	0.18117537	0.119702123	0.34844768
hsa.miR.33a	0.2882977	0.1860548	0.121254113	0.34844768
hsa.miR.186	0.30698336	0.1983606	0.121718025	0.34844768
hsa.miR.876.3p	-0.3666957	0.2404392	0.127232185	0.36069707
hsa.miR.425	0.33544388	0.22066848	0.128479318	0.36073039
hsa.miR.22	-0.4172473	0.2773605	0.132491251	0.36845186
hsa.miR.2054	0.25075535	0.17265607	0.146407029	0.40268867
hsa.miR.29c	0.38246608	0.26411775	0.147592088	0.40268867
hsa.miR.320c	-0.2515403	0.17428296	0.148939644	0.40268867
hsa.miR.27a	-0.2415154	0.16878164	0.152449044	0.40839561
hsa.miR.122	0.50397608	0.35466646	0.15532082	0.4118755
hsa.miR.450b.5p	0.2576432	0.18186083	0.156569112	0.4118755
hsa.miR.346	-0.2430956	0.17228397	0.158239677	0.41255344
hsa.miR.2114	0.24101837	0.17188183	0.160845702	0.41563668
hsa.let.7c	-0.2971	0.21269294	0.162458853	0.41612268
hsa.miR.25	0.3189414	0.23110152	0.167558263	0.42439258
hsa.miR.145	-0.2510241	0.18233295	0.168594313	0.42439258
hsa.miR.194	0.23953406	0.17638572	0.174459629	0.43266737
hsa.miR.370	-0.2594902	0.19125174	0.174845035	0.43266737
hsa.miR.2117	-0.2201892	0.16284735	0.176336914	0.43269226
hsa.miR.523	0.25795434	0.19264339	0.1805625	0.43884784
hsa.miR.448	-0.2826391	0.21170259	0.181851329	0.43884784
hsa.miR.548h	0.27989716	0.21072355	0.184089884	0.44060858
hsa.miR.375	0.25263089	0.19117941	0.186356843	0.44240812
hsa.miR.544	-0.2670415	0.20606642	0.195009951	0.45921698
hsa.miR.494	-0.2475085	0.19496578	0.204263736	0.47716009
hsa.miR.21	0.22484252	0.18251752	0.217988098	0.50517877
hsa.miR.518f	0.23340608	0.1916091	0.223172014	0.50949471
hsa.miR.151.3p	0.21459184	0.176228	0.223340147	0.50949471
hsa.miR.99a	0.33710404	0.2792852	0.227422771	0.51333122
hsa.miR.374b	-0.2770902	0.23011576	0.228537874	0.51333122
hsa.miR.381	-0.2072885	0.17671263	0.240785289	0.53671225
hsa.miR.592	0.39382987	0.33928355	0.245735799	0.54359737
hsa.miR.1979	-0.1998258	0.17495261	0.253382928	0.55629936
hsa.miR.363	0.27637372	0.24413672	0.257615578	0.56137126
hsa.miR.491.5p	-0.2326067	0.20751441	0.262322538	0.56739393
hsa.miR.548a.3p	0.20580287	0.18590021	0.26826758	0.57598628
hsa.miR.548j	0.20579089	0.18832919	0.274517183	0.58510232
hsa.miR.95	0.3082369	0.28349772	0.276920093	0.58594686

hsa.miR.10b	-0.2058838	0.1906573	0.280203134	0.58691171
hsa.miR.490.5p	0.2120838	0.19743474	0.282734418	0.58691171
hsa.miR.93	-0.256314	0.23894298	0.283405996	0.58691171
hsa.miR.1275	0.16409948	0.15576416	0.292106219	0.60002966
hsa.miR.488	-0.2417977	0.23131679	0.295879789	0.60002966
hsa.miR.423.5p	0.26880221	0.25716419	0.295905039	0.60002966
hsa.miR.454	-0.2291959	0.22099865	0.299692967	0.60062114
hsa.miR.376b	0.18722385	0.18075855	0.300310569	0.60062114
hsa.miR.551b	0.21708708	0.21064754	0.302742412	0.60136588
hsa.miR.424	-0.1979267	0.19344716	0.306233865	0.60419114
hsa.miR.329	-0.2268505	0.22501947	0.313388628	0.61415758
hsa.miR.219.2.3p	0.20116123	0.20060684	0.315974946	0.6150979
hsa.miR.30a	0.27459256	0.27679839	0.321182447	0.62109453
hsa.let.7e	-0.3414931	0.34910533	0.327977932	0.62821802
hsa.miR.144	0.12205365	0.12508201	0.32916903	0.62821802
hsa.miR.190b	-0.1759324	0.18115538	0.331464436	0.62849101
hsa.miR.151.5p	0.19876281	0.20635422	0.335441274	0.63192808
hsa.miR.520d.5p_hsa.miR.527_hsa.miR.518a.5p	-0.186328	0.19844051	0.347750594	0.65091778
hsa.miR.450a	0.19283431	0.20751851	0.352765197	0.65609833
hsa.miR.361.3p	-0.2703621	0.29417837	0.358073966	0.66175695
hsa.miR.9	0.2365937	0.25889168	0.360784417	0.66257264
hsa.miR.143	-0.1373046	0.15119163	0.363799117	0.66393339
hsa.miR.580	0.16796776	0.19305623	0.384275383	0.69461455
hsa.miR.130b	-0.1938794	0.22335114	0.385368344	0.69461455
hsa.miR.132	-0.1435542	0.16715994	0.390459881	0.69947414
hsa.miR.23a	0.21241599	0.250367	0.396204945	0.70321813
hsa.miR.149	0.14355806	0.1696234	0.397366406	0.70321813
hsa.miR.136	0.16289265	0.19429333	0.401814407	0.70680607
hsa.miR.504	-0.2158252	0.25921629	0.405066693	0.70772334
hsa.miR.500_hsa.miR.501.5p	0.17516932	0.21133749	0.407183292	0.70772334
hsa.miR.584	0.16026383	0.19461907	0.410238253	0.70881402
hsa.miR.345	0.17972631	0.22157128	0.41728275	0.71597036
hsa.miR.223	0.16687046	0.20762076	0.421554537	0.71597036
hsa.miR.452	0.14753512	0.18478664	0.424633821	0.71597036
hsa.miR.153	-0.1385893	0.17362544	0.424749495	0.71597036
hsa.miR.34c.5p	0.17137746	0.21558142	0.42663987	0.71597036
hsa.miR.100	0.23899882	0.30500409	0.43327945	0.72295771
hsa.miR.98	0.14428113	0.18780247	0.442332731	0.72723564
hsa.miR.1225.3p	0.16016588	0.20868351	0.442780621	0.72723564
hsa.miR.487a	0.15961431	0.20820862	0.443314875	0.72723564
hsa.miR.563	0.19302752	0.25543908	0.449847385	0.72961311
hsa.miR.577	0.14241862	0.18852271	0.449981784	0.72961311
hsa.miR.655	-0.1898566	0.25360384	0.454077502	0.72961311
hsa.miR.129.3p	-0.1596487	0.21357545	0.454758858	0.72961311
hsa.miR.342.3p	0.21084301	0.29420541	0.473588615	0.75567145
hsa.miR.377	0.1682142	0.24260002	0.488070581	0.77454679

hsa.miR.1537	0.13356278	0.19508095	0.493562847	0.77661496
hsa.miR.127.3p	-0.1056365	0.15469639	0.494693089	0.77661496
hsa.miR.185	-0.1274038	0.18900608	0.500265084	0.77911606
hsa.miR.137	-0.1415941	0.21305977	0.506322579	0.77911606
hsa.miR.522	-0.1126848	0.16970721	0.506693376	0.77911606
hsa.miR.379	-0.2118221	0.320663	0.508884634	0.77911606
hsa.miR.99b	0.14985879	0.22725913	0.509627288	0.77911606
hsa.miR.34b	0.12810071	0.19649039	0.514437368	0.7823735
hsa.miR.431	-0.1263026	0.19537407	0.517978034	0.78367661
hsa.miR.497	-0.1518708	0.23764377	0.522777895	0.78686157
hsa.miR.187	-0.1286002	0.20295679	0.526320008	0.78813047
hsa.miR.331.3p	-0.1425214	0.22996105	0.535413722	0.79765718
hsa.miR.615.5p	-0.1042346	0.17359596	0.548210726	0.81208015
hsa.miR.126	0.13842609	0.23245569	0.551512948	0.81208015
hsa.miR.453	0.11410462	0.19254395	0.553438184	0.81208015
hsa.miR.139.5p	-0.1744387	0.29850835	0.558972894	0.81391867
hsa.miR.627	0.11461479	0.19757934	0.561850563	0.81391867
hsa.miR.485.3p	-0.144761	0.25084485	0.563876209	0.81391867
hsa.miR.626	-0.1215395	0.21279672	0.567895861	0.81391867
hsa.miR.200a	0.07477245	0.13132932	0.56911811	0.81391867
hsa.miR.539	0.20081026	0.35480848	0.571415502	0.81391867
hsa.miR.103	0.13179638	0.23694889	0.578058546	0.81843955
hsa.miR.708	0.11810568	0.21353418	0.580195161	0.81843955
hsa.miR.337.3p	-0.1038031	0.19591826	0.596230808	0.83305983
hsa.miR.600	0.10675084	0.20502253	0.602590701	0.83305983
hsa.miR.548f	0.08458593	0.16297103	0.603743788	0.83305983
hsa.miR.148a	0.1112885	0.21469798	0.60421475	0.83305983
hsa.miR.140.3p	-0.1146987	0.22385639	0.608387607	0.83305983
hsa.miR.582.5p	-0.1277525	0.2522818	0.612584374	0.83305983
hsa.miR.451	0.06424753	0.12711172	0.613248831	0.83305983
hsa.miR.133b	-0.0878572	0.17388854	0.613383097	0.83305983
hsa.miR.193b	-0.1040684	0.20899823	0.618526962	0.83341325
hsa.miR.27b	0.20147419	0.40556879	0.619351627	0.83341325
hsa.miR.106a_hsa.miR.17	0.11566628	0.23834737	0.627474567	0.84047052
hsa.miR.339.3p	0.08996811	0.19359189	0.642124262	0.85616568
hsa.miR.515.3p	0.08445484	0.18427719	0.646734591	0.85699749
hsa.miR.496	-0.093977	0.207549	0.650697026	0.85699749
hsa.miR.24	-0.1185449	0.26493544	0.654551486	0.85699749
hsa.miR.204	-0.0838402	0.18962333	0.658387359	0.85699749
hsa.miR.1260	-0.0486253	0.11025412	0.659191941	0.85699749
hsa.miR.495	-0.0926296	0.21216203	0.662402744	0.85699749
hsa.miR.517c_hsa.miR.519a	0.08797095	0.20205936	0.663292578	0.85699749
hsa.miR.1305	-0.0700004	0.1628229	0.66725574	0.85832016
hsa.miR.548a.5p	-0.0879397	0.20955142	0.67473603	0.86210962
hsa.miR.660	0.09824672	0.23516265	0.676106513	0.86210962
hsa.miR.766	0.08241322	0.20194055	0.683195618	0.86553493
hsa.miR.340	-0.0974856	0.24051534	0.685242568	0.86553493

hsa.miR.221	0.09950861	0.24753491	0.687685283	0.86553493
hsa.miR.106b	-0.0892815	0.24498968	0.715537081	0.89654956
hsa.miR.1977	-0.0607013	0.1683775	0.718467799	0.89654956
hsa.miR.101	-0.0732697	0.21179938	0.729388356	0.90630383
hsa.miR.326	0.06007283	0.18256599	0.742120022	0.9182163
hsa.miR.526a_hsa.miR.518d.5p_hsa.miR.520c.5p	-0.0642114	0.21711643	0.767423724	0.9336988
hsa.miR.767.5p	0.05885276	0.20330671	0.772215841	0.9336988
hsa.miR.146b.5p	0.06527903	0.22889651	0.775498458	0.9336988
hsa.miR.934	0.05735821	0.20115914	0.775538067	0.9336988
hsa.miR.10a	0.07170771	0.25356304	0.777329919	0.9336988
hsa.miR.301a	0.07330667	0.2613395	0.77909118	0.9336988
hsa.miR.92a	0.07661425	0.28101242	0.785132624	0.9336988
hsa.miR.1297	0.05247665	0.19705862	0.790008424	0.9336988
hsa.miR.208a	0.04689492	0.17675058	0.790765321	0.9336988
hsa.miR.744	-0.0439282	0.16799908	0.793723467	0.9336988
hsa.miR.520d.3p	-0.051053	0.20209605	0.800563959	0.9336988
hsa.let.7a	0.04569716	0.18531193	0.805220988	0.9336988
hsa.miR.1185	0.05841252	0.24088261	0.808397641	0.9336988
hsa.miR.199b.5p	-0.0439065	0.18166367	0.809019519	0.9336988
hsa.miR.656	0.06399358	0.26976214	0.812484382	0.9336988
hsa.miR.874	-0.0405931	0.17499019	0.816558655	0.9336988
hsa.miR.598	-0.0776761	0.33980146	0.819185652	0.9336988
hsa.miR.362.3p	0.0505075	0.22141063	0.819555397	0.9336988
hsa.miR.382	0.0448082	0.19659688	0.819709072	0.9336988
hsa.miR.96	-0.0615672	0.27441999	0.82248203	0.9336988
hsa.miR.31	0.05554765	0.2534385	0.826512921	0.9336988
hsa.miR.199a.5p	-0.0368799	0.1698366	0.828091916	0.9336988
hsa.miR.365	-0.0397631	0.18320722	0.828178045	0.9336988
hsa.miR.1299	-0.0295563	0.14233541	0.835500067	0.93833084
hsa.miR.509.3p	-0.0323149	0.15909859	0.839047066	0.938704
hsa.miR.320a	0.04052342	0.20677456	0.844626805	0.9413398
hsa.miR.181a	-0.0441799	0.27874517	0.874066373	0.96717456
hsa.miR.219.5p	-0.0196961	0.12550904	0.875300251	0.96717456
hsa.miR.1	0.0320444	0.20831089	0.877744033	0.96717456
hsa.miR.199a.3p_hsa.miR.199b.3p	0.029957	0.20629935	0.884544036	0.97100323
hsa.miR.603	0.02400209	0.17388467	0.890213121	0.97155798
hsa.miR.181c	0.04156224	0.30527143	0.891703898	0.97155798
hsa.miR.152	-0.0221594	0.1737382	0.898509044	0.97532564
hsa.let.7i	0.04060835	0.32925369	0.9018422	0.97532564
hsa.miR.188.5p	0.01813239	0.18863191	0.923420663	0.99376104
hsa.miR.590.5p	-0.0234771	0.29716793	0.937030361	0.99376104
hsa.miR.195	-0.0162449	0.23014336	0.943727422	0.99376104
hsa.miR.516a.3p	-0.0126787	0.18233064	0.944562096	0.99376104
hsa.miR.514	-0.0114851	0.16962838	0.946018612	0.99376104
hsa.miR.432	0.01861321	0.28656682	0.948211876	0.99376104
hsa.miR.595	-0.01221	0.21988635	0.955717242	0.99376104

hsa.miR.29a	-0.0150584	0.2768515	0.956623048	0.99376104
hsa.miR.324.5p	0.01216005	0.23782781	0.959222233	0.99376104
hsa.miR.520e	-0.0067663	0.13443826	0.959859425	0.99376104
hsa.miR.107	0.01024586	0.25809789	0.968334247	0.99376104
hsa.miR.16	-0.0088474	0.25056923	0.971833157	0.99376104
hsa.miR.206	0.00759115	0.21981103	0.972450604	0.99376104
hsa.miR.1286	0.00592031	0.17883861	0.973591491	0.99376104
hsa.miR.542.3p	-0.0051861	0.18852594	0.978054084	0.99376104
hsa.miR.579	0.0044635	0.17848191	0.980048457	0.99376104
hsa.miR.135a	0.00479346	0.20259621	0.981123701	0.99376104
hsa.miR.146a	-0.0080087	0.34695576	0.981584254	0.99376104
hsa.miR.34a	0.00399646	0.19384273	0.983551166	0.99376104
hsa.miR.140.5p	-0.0010755	0.28031116	0.996938754	0.99894955
hsa.miR.330.3p	0.00056747	0.16512765	0.997258037	0.99894955
hsa.miR.873	0.00031513	0.23935908	0.998949548	0.99894955

Supplementary Table 2: Genes that are common between predicted targets of miR-484 and BLSA protein co-expression module M1 blue

ARL15
ATP6V1D
SEMA4D
CACNA1E
SEPT3
ABR
CPLX2
WDFY1
SLC25A27
CNRIP1
C2CD2L
GPD2
PHYHIP
BAIAP2
RTN3
SH3GLB2
DBNL
ATP6V1F
SH3GL1
SV2B
HPCAL1
LASP1
GABBR2
VAMP2
RAB3D
CRMP1
GNB1
AP1G1
RAPGEF4
PIP5K1C
ADD2
LINGO1
CAPN5
PCDH1
STMN3
AP1S1
GIT1
ARMC10
NCAM1
TLN2
GNG4
CAPZA2

PAM
PGM2L1
NCKAP1
MCTS1
